# Distinct monitoring strategies underlie costs and performance in prospective memory

**DOI:** 10.3758/s13421-022-01275-5

**Published:** 2022-04-06

**Authors:** Seth R. Koslov, Landry S. Bulls, Jarrod A. Lewis-Peacock

**Affiliations:** 1 Department of Neurosurgery, Perelman School of Medicine at the University of Pennsylvania, 3700 Hamilton Walk, Philadelphia, PA 19104 USA; 2 Department of Psychology and Brain Sciences, Dartmouth College, Hanover, NH USA; 3 Department of Psychology, University of Texas at Austin, Austin, TX USA

**Keywords:** Prospective memory, Eye movements, Attention, Memory

## Abstract

Prospective memory (PM) describes the ability to remember to perform goal-relevant actions at an appropriate time in the future amid concurrent demands. A key contributor to PM performance is thought to be the effortful monitoring of the environment for PM-related cues, a process whose existence is typically inferred from a behavioral interference measure of reaction times. This measure, referred to as “PM costs,” is an informative but indirect proxy for monitoring, and it may not be sufficient to understand PM behaviors in all situations. In this study, we asked participants to perform a visual search task with arrows that varied in difficulty while concurrently performing a delayed-recognition PM task with pictures of faces and scenes. To gain a precise measurement of monitoring behavior, we used eye-tracking to record fixations to all task-relevant stimuli and related these fixation measures to both PM costs and PM accuracy. We found that PM costs reflected dissociable monitoring strategies: higher costs were associated with early and frequent monitoring while lower costs were associated with delayed and infrequent monitoring. Moreover, the link between fixations and PM costs varied with cognitive load, and the inclusion of fixation data yielded better predictions of PM accuracy than using PM costs alone. This study demonstrates the benefit of eye-tracking to disentangle the nature of PM costs and more precisely describe strategies involved in prospective remembering.

Imagine you’re on a road trip, and you pass a billboard advertising a roadside stop serving “the best bar-b-que” in the state. You form an intention to stop and eat there, but how do you remember to follow through with this delicious plan? You likely won’t be able to continuously linger on the plan, because your attention will be needed to perform other urgent tasks, such as negotiating a patch of heavy traffic, processing map instructions, or calming a child in the backseat. Prospective memory (PM) describes our ability to perform future intentions at a specific time despite concurrent demands from the environment. The multiprocess framework of prospective memory (Einstein & McDaniel, 2005) describes two dissociable strategies that individuals use to perform prospective intentions, referred to as *active monitoring* and *spontaneous retrieval*. Active monitoring is typically characterized by two components: the allocation of attention towards effortful monitoring of the environment for cues to perform the prospective intention and sustained representation of the intention (Ballhausen et al., 2017; Guynn, 2008; McDaniel & Einstein, 2000). For example, while driving, you may be checking road signs for an indication that the exit for the restaurant is ahead, while also keeping the name of the bar-b-que spot in mind. On the other hand, spontaneous retrieval is characterized by the encoding of cue–action associations in episodic memory and reliance on cues in the environment to trigger the retrieval of the intention when it is needed (Harrison et al., 2014; Rummel & Meiser, 2013; Scullin et al., 2010). For example, you may continue driving without thinking about your appetite and then happen to read a sign that suddenly makes you remember, “Don’t forget about that bar-b-que!.” While it is evident that there are significant individual and group differences in how effectively individuals engage these two strategies (Ball et al., 2020; Brewer et al., 2010), precise descriptions of how they are implemented remain elusive.

The multiprocess theory of PM posits that active monitoring, a component of *proactive control*, is an effortful process that draws resources from a shared-capacity system split between ongoing demands and future intentions. Because cognitive resources must be split between a PM task and other ongoing demands, individuals may vary in how and when they allocate resources for monitoring (e.g., Scullin et al., 2013). For example, Marsh et al. (2006) found that when participants were aware that PM targets would only appear during a specific portion of the experiment, they only monitored during those times. Situational-specific monitoring has also been observed when the probability of a prospective intention is high (Bugg & Ball, 2017; Cohen et al., 2017; Kuhlmann & Rummel, 2014; Smith et al., 2017), when the intention is deemed sufficiently important (Loft & Yeo, 2007; Smith & Hunt, 2014), or when the perceived difficulty of performing the intention is high (Lourenço et al., 2015; Rummel & Meiser, 2013). When monitoring ability is impaired due to high ongoing cognitive load (Marsh et al., 2002; Marsh et al., 2005; Meier & Zimmermann, 2015), PM performance can suffer. Thus, a bevy of previous research suggests that monitoring is a resource-demanding task, and that adapting to environmental demands involves shifting the amount of resources devoted towards monitoring. Conversely, individuals have the ability in some situations to stop monitoring and instead offload prospective intentions to episodic memory (i.e., to engage *reactive control*) without sacrificing PM performance (Anderson et al., 2019; Koslov et al., 2019; Lewis-Peacock et al., 2016). To capture this nuance, the dynamic multiprocess view of PM (DMPV; Scullin et al., 2013; Shelton & Scullin, 2017) updated the original multiprocess theory (Einstein & McDaniel, 2005) to specify that the use of proactive or reactive control depends on environmental and contextual factors. What is less clear to researchers, is exactly how individuals adjust these cognitive resources in order to carry out PM strategies.

The most prominent method for determining when individuals are monitoring for prospective intentions, and to generally infer processes that may underlie monitoring, is to use a dual-task interference metric. This interference metric is called “PM costs” and is a measure of response slowing that occurs on an ongoing task when participants are asked to concurrently perform a PM task. These costs are posited to reflect the amount of cognitive resources that are devoted to actively monitor for cues related to PM intentions (Smith, 2003). Larger costs are thought to indicate more resources being allocated towards the prospective intention and can relate to a greater ability to notice and act on prospective intentions (e.g., Anderson et al., 2019; Koslov et al., 2019). Sometimes there are no PM costs observed (Harrison et al., 2014; Rummel & Meiser, 2013; Scullin et al., 2010), and in such cases it is commonly assumed that participants are relying on spontaneous retrieval or some reactive control process rather than active monitoring (Braver, 2012). Of course, the absence of evidence of PM costs does not constitute evidence for the absence of active monitoring. PM costs may not capture all forms of monitoring behaviors, and they are likely influenced by other sources, including maintaining an intention in working memory and inhibiting ongoing responses. A more precise explanation of the processes that underlie PM costs and how they relate to both strategy and performance is warranted.

One approach for understanding the nature of PM costs has been to use data aggregation and computational modeling to dissect RTs into more precise components (for review, see Strickland et al., 2019b). For example, researchers have fit RT distributions with ex-Gaussian functions in order to attempt to distinguish between evidence for continuous monitoring, as represented by the *μ* parameter, and sporadic monitoring, as represented by the *τ* parameter (Ball et al., 2015; Loft et al., 2014). Results indicate that PM costs may be related to both transient and sustained monitoring or “checking” processes, although PM task accuracy was found to be more closely linked to the continuous monitoring component (Ball & Brewer, 2018). Another computational approach has been to use evidence accumulation models to identify decision-making processes that likely contribute to PM costs. These models have been used to measure the relative impact of decision thresholds, indicating how much evidence must be collected before an individual will make a response, versus evidence accumulation rates that indicate how quickly information about the task can be collected. In short, it follows from the shared-capacity explanation of PM costs from the multiprocess views that evidence accumulation rates to ongoing tasks should be slowed when individuals have prospective intentions to perform. However, most studies using evidence-accumulation modeling have found that ongoing task decision thresholds, not accumulation rates, change as a function of prospective intentions (e.g., Heathcote et al., 2015; Horn & Bayen, 2015; Strickland et al., 2017). In other words, these studies have supported the claim that PM costs result when individuals delay or take a more cautious approach to the ongoing task when also monitoring for prospective intentions (Loft & Remington, 2013).

The “delay theory” interpretation of PM costs is controversial, and other researchers have suggested that threshold adjustments do not sufficiently explain task performance in many circumstances. Using an extension of classic accumulation models, referred to as the Prospective Memory Decision Control (PDMC) architecture, researchers have found that both delay and shared-capacity explanations can explain PM costs, depending on the ongoing demands of the environment (Boag et al., 2019; Strickland et al., 2019a). Additionally, Anderson and McDaniel (2019) recently tested predictions from the delay theory of PM costs by comparing PM performance in a condition that biased participants towards longer ongoing task latencies (i.e., longer delay periods) versus a condition that biased participants towards shorter latencies. Contrary to predictions from the delay theory, PM performance was greater in the shorter latency condition. At present, there is no consensus on the mechanisms that drive PM costs and subsequent PM performance. One limitation to the modeling approach is that it requires aggregation of large numbers of trials, making it difficult to analyze processes that may be happening over short intervals, such as brief changes in monitoring strategies in response to cues or distractions in the environment. Additionally, while many accumulation models can successfully predict PM costs, they often struggle to explain variance in PM accuracy. It has been suggested that changes in ongoing task decision thresholds may not have a strong impact on PM performance (Strickland et al., 2019b). It is also possible that a link between PM costs and PM performance may only exist under certain conditions (Anderson et al., 2019; Koslov et al., 2019), and this relationship needs more careful observation and modeling to understand (Strickland et al., 2018, 2019a, 2020).

One promising approach is eye-tracking, which provides direct measurement of the allocation of visual attention with excellent spatiotemporal precision, thus allowing tracking of monitoring during PM task performance. Whereas aggregating over trials improves statistical power, eye-tracking affords the ability to observe precise moments in time to identify monitoring behaviors across different situations and individuals. To date, only a handful of studies have used eye-tracking to study PM (e.g., Ballhausen et al., 2019; Bowden et al., 2017; Chen et al., 2013; Hartwig et al., 2013; Shelton & Christopher, 2016; West et al., 2007), and these have provided unique insights into monitoring behavior. In general, these studies support the idea that individuals increase attention allocated towards active monitoring of PM stimuli in response to environmental cues or contexts, and that increased active monitoring is related to better PM performance. For example, Hartwig et al. (2013) measured fixation patterns while participants performed targeted visual search, viewed scenes freely, or viewed scenes with a prospective intention to indicate when specific objects appeared. Fixation patterns on PM misses (when participants failed to identify the prospective object) were most similar to those observed during free viewing, while on PM hits the fixation patterns were closer to those observed during directed visual search. Shelton and Christopher (2016), found that when individuals were reminded of a PM intention by semantically related cues, they were more likely to monitor for and complete that intention. Kalpouzos et al. (2010) found different visual search patterns before versus after performing a prospective intention, such that a participant’s gaze covered more distance while searching for a PM target than after it was found. Bowden et al. (2017) observed that when participants were informed that a possible PM event was likely to occur soon, they increased fixations on PM-related information. These studies provide direct evidence that active monitoring increases when a PM event is likely, and that increased monitoring can improve PM accuracy. However, none have attempted to link direct measures of monitoring from eye-tracking to the more commonly used but indirect measures of monitoring from PM costs.

Here, we used eye-tracking to relate monitoring behavior to both PM costs and PM accuracy. The standard assumptions are that PM costs consistently reflect active monitoring, and that monitoring is beneficial for PM performance. However, recent evidence shows that the link between costs and performance depends on the demands of the task (Koslov et al., 2019). This context-dependent relationship suggests that PM costs may not always reflect the frequency of monitoring but are influenced by the *nature* of monitoring (e.g., the combination of timing, duration, and frequency of fixations on PM stimuli) in a particular context. We investigated and characterized the association between PM costs and active monitoring across different levels of PM costs and environmental demands. We next examined whether fixation measures provided additional information, above and beyond PM costs, for explaining PM accuracy. To preview our results, we found that monitoring strategies are diverse and dependent on environmental demands, and that PM costs, while useful, only tell part of the story of monitoring behavior and PM performance.

## Materials and methods

Participants (*N* = 30, 17 females, mean age = 18.8 years) were recruited from the University of Texas at Austin undergraduate community to participate in the experiment. We collected this sample size for two reasons. Our first concern was ensuring that we had sufficient power to detect changes in PM costs that would correspond to different levels of ongoing task difficulty. To estimate this, we looked at data previously collected from our lab to determine the effect size of the difference in PM cost between easy and hard difficulty levels. The effect size, using a paired-sample *t* test, was 0.767. Thus, for an alpha of 0.05 and to achieve power (1 − beta) of at least 0.95, we would need a sample size of at least *N* = 25. Next, we referenced the PM literature where eye-tracking was used, which ranged between *N* = 7 to 32 participants. Thus, we choose a sample size of *N* = 30, which is in line with prior published work (Koslov et al., 2019).

Participants were given 2 hours of experiment credit in exchange for participation. After obtaining informed consent, participants were asked to place their heads on a chin rest, sitting 18 inches from the computer screen, in a position that may be as comfortable as possible for the duration of the experiment. The experiment took place in a dimly lit room to limit light interference. The experimenter adjusted the eye-tracker camera in order to get the best pupil and corneal reflection recognition possible. Once the camera had been adjusted, 5-point calibration and validation procedures were used to make sure that fixations in the vertical and horizontal directions were being accurately tracked. Automatic adjustment of corneal and pupil thresholds was almost always sufficient for good eye-tracking, but occasionally, manual adjustments were made to improve the calibration. Once calibration was successfully performed, participants continued to the experiment instructions.

### Eye-tracking details

Eye-tracking data was collected using the SR Research EyeLink 1000 plus desktop mount and then converted to an ASCII file. Samples were collected at 250 Hz (*N* = 17) or 500 Hz for others (*N* = 13) and down-sampled to 250 Hz, and no differences were found in dwell time measurements between sampling rates (*p* = .647). Data were recorded for the left eye from all participants unless calibration was not possible with that eye. For *N* = 2 participants, calibration was more successful for right eye than the left eye, and so was used for data collection instead. The left eye was chosen as the default simply in order to keep the eye-link apparatus in as similar a position as possible for all participants, to standardize easy-to-follow experiment protocols, and to reduce the time necessary for fixation validation and recalibration. Drift correction was performed throughout the experiment, at the rate of once every five trials. Drift correction values were not used as an automatic correction to fixation locations, but instead they were used as a way of checking the quality of the fixation calibration. If the fixation location was more than approximately 0.5° from the fixation target, recalibration and validation were performed. No further corrections were applied to the eye-tracking data. During data collection, calibration for three participants was either never successfully achieved or extremely variable over the course of the experiment. Data for those participants was collected but subsequently excluded from the final sample, and three new participants were included in the study, resulting in the *N* = 30 sample analyzed here. Default SR Research EyeLink parsing parameters were used to determine saccades, fixations, and blinks, and eye movements with a velocity exceeding 35 degrees/s and an acceleration exceeding 8,000 degrees/s^2^ were tagged as saccades.

### Ongoing task details (OG task)

**Fig. 1:**
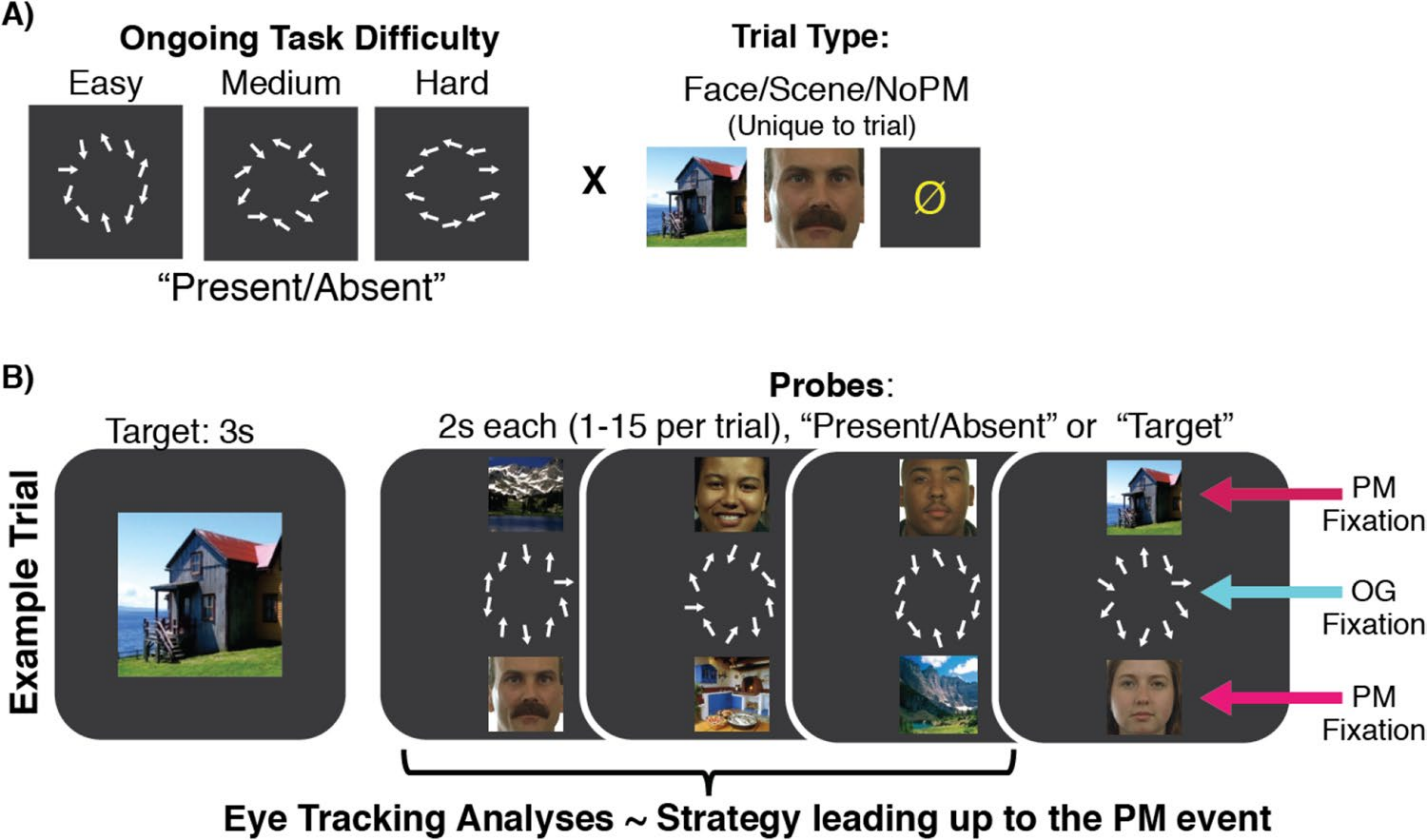
**a** On each trial, the ongoing task would either be easy, medium, or hard, depending on the similarity of the distractor arrows to the target arrow as depicted here. Each trial began with a unique face or scene target (PM trials), or a null symbol (non-PM trials). **b** Trial design. A target image was displayed for 3-s. After a 1-s delay, participants saw a series of 1–15 probes on the screen for 2-s each. If the PM-target reappeared, it did so on the last probe of the trial. Fixations were determined to fall within the PM-target ROIs if they were within 15 pixels (0.58°) of the boundaries of either of the PM-stimuli. The arrow array ROI extended in 110 pixels (4.22°) in every direction from the center of the screen

The experiment was conducted using MATLAB with Psychtoolbox (Version 3; Brainard, 1997), using a modified version of the dual-task PM paradigm developed in Koslov et al. (2019). Participants were asked to perform a visual search task where they indicated the absence or presence of a rightward-facing horizontal arrow (⇨) in an array of 10 distractor arrows (see Fig. 1a). Participants sat approximately 18 inches away from the screen, and all 10 arrows, which were 1.04° by .22° in shape, were presented at 3.18° away from the center of the screen. This OG task was chosen because it allows for the ability to systematically and parametrically manipulate task difficulty by adjusting distractor parameters along a continuum (Kiyonaga et al., 2017; Koslov et al., 2019; Sobel et al., 2007). The target arrow was present on only a randomly selected half of the trials, located in one of 10 semirandomly selected possible locations around the circle. Participants were instructed to search for the target on each display and use their left hand to press “1” for present and “2” for absent. Target arrow location was counterbalanced between the top and bottom halves of the screen. On “target-present” displays, nine nontarget (distractor) arrows appeared in set positions around the circular array (10 on “target-absent” displays), oriented within some distribution of angles determined by the current task difficulty setting. OG task difficulty was manipulated on each probe by adjusting two parameters that determined the orientation of the distractor arrows: their minimum and maximum similarity to the target arrow (horizontal). The OG task could have a difficulty level of either *easy* (minimum 45° from horizontal, maximum 85° from horizontal), *medium* (min 25°, max 45°), or *hard* (min 15°, max 25°). On every search display, each distractor arrow had a 50% chance of being flipped across the horizontal plane and a 50% chance of being flipped across the vertical plane, so that arrows could possibly cover the whole 360° continuum. In previous pilot testing, we validated that accuracy decreased and reaction time increased as the OG task became more difficult, while accuracy remained below ceiling and above floor (see Koslov et al., 2019).

### PM task stimuli

Each probe contained both a visual search array (the OG task) and a single face and a single scene image (the PM task). PM task stimuli consisted of colored images of unfamiliar faces and unfamiliar scenes which were gathered from various in-house and online sources. These images were controlled for valence and familiarity (see Lewis-Peacock et al., 2016). For each participant, 60 faces and 60 scenes were randomly selected to serve as the PM targets, and 100 faces and 100 scenes were used as distractors. PM target images did not appear as distractors and were used on one trial only. Distractor images never reappeared within the same trial, but later reappeared on subsequent trials (mean exposures per distractor = 14, min = 13, max = 15). Multiple repetitions of distractor images across trials allowed for increased novelty of PM target images. PM stimuli (each of size 11.5° × 11.5° in visual angle) were horizontally aligned centrally and vertically aligned with the middle of the images placed 11.5° above or below the OG task search array (meaning the nearest image edge was 5.75° away from the center of the screen or ~2.5° away from the center of the nearest OG task arrow). The proximity and size of the image targets to the arrow array meant that information from the face and scene images was peripherally available to participants, however not within foveal center. While salient information about the face and scene images could be perceived via covert or peripheral attention, fixations toward the images were necessary to bring them into high-precision representation in the foveal center.

### Dual-task PM experiment design

Each trial began with the presentation of the PM target (“PM-trials”: a face or a scene) or a yellow null (∅) sign (no-PM trials) for 3 s, followed by a 1-s fixation cross. On PM trials, participants were informed that the PM target shown was only relevant for the current trial. PM trials were further designated as PM-present or PM-absent trials. On PM-present trials, the PM target reappeared once, while on PM-absent trials, it did not. We included PM-absent trials to limit the increasing expectancy of a PM target appearing as trial length increased. This design led to 15 total trial types (PM trials: face/scene × easy/medium/hard × present/absent; non-PM trials: easy/med/hard), which were randomly interleaved so that participants had one of each trial type in each block.

Trials were semi-randomly designated to be between 1 and 15 probes in length (mean = 9.3 probes). Each probe was on the screen for 2-s, during which one face, one scene, and one arrow array were presented simultaneously on the screen. Participants were allowed 1.9 s to respond to the presence or absence of the horizontal arrow in the OG task, or to indicate whether the PM target had reappeared (by pressing “3”). Participants were instructed to equally weight the importance of both tasks, and only one response (to either the OG task or the PM task) was allowed per probe (“task-switch” approach; Bisiacchi et al., 2009). Visual feedback was presented immediately following each response, in the form of the arrows turning green for correct OG task responses, turning red for incorrect OG task responses, or a yellow border surrounding the screen for PM false alarms. Probe feedback remained on screen for the remaining duration of each 2-s probe. The 1.9-s response deadline ensured that some time (minimum 100 ms) was always devoted to feedback on every probe. On PM-present trials, the PM target always reappeared on the last probe of that trial. Participants were given feedback on the PM task in the form of a green border appearing around the edge of the screen for correct PM responses and a red border for missed PM targets, or a fixation cross for PM-absent trials. This feedback remained for 2-s and was followed by a 6-s ITI with a fixation cross on the screen before the next trial began. On non-PM trials, participants were instructed to ignore the face and scene images and focus solely on the OG task. At the end of these trials, participants saw the fixation cross for 2 s to indicate the end of the trial, followed by a 6-s ITI. Feedback was provided at the end of each trial to ensure that participants knew that the previous PM target was no longer relevant.

Participants performed one trial of each of the 15 unique trial types on each of the 10 blocks of the experiment. Trial order and trial lengths were semirandomly mixed for each block, so that there was no predictable relationship between trial types or trial duration. Between three to five trials on each block (40 per participant across the entire experiment) were semirandomly assigned to be shorter than eight probes in length and were considered “catch trials” and were excluded from analysis. These trials were included so that participants were aware that the PM target could reappear at any time. The task included 1,340 total probes, with approximately the same number of probes for each trial type. There were 60 total PM events, thus approximately 4.5% of probes contained a PM target.

During the first two blocks of the experiment, participants were encouraged to ask clarifying questions about the experiment. These first two blocks were treated as practice and were not included in the analysis. Participants then performed eight more experimental blocks. At the end of each block, participants were given feedback on their overall PM task accuracy and OG task accuracy. If accuracy was low on either task (below chance, or 50%, on the OG task and below 33% for the PM task), participants were verbally encouraged to try harder on the next block. When participants indicated that they were ready to continue with the experiment, recalibration and validation of the eye tracker was performed before proceeding to the next block.

### Measuring monitoring

Monitoring was quantified in two ways. First, we measured the total time gazing at PM stimuli and at the OG task array on each probe, between the start of the probe and the time that a response was made. These gaze times are referred to as the *cumulative PM-dwell time* and *cumulative OG-dwell time*, respectively (see Fig. 1b). Secondly, for every 4-ms sample, we calculated the Euclidean distance of each fixation from the center of the screen (Fig. 3a–b). The edge of the arrows in the OG task’s array extended to 110 pixels (4.22°) from the center of the screen, while the PM stimuli began at 150 pixels (5.75°) from the center of the screen. To control for variable response times across probes, we scaled the time-axis of these data so that 0 indicated the start of the probe and 1 indicated the response time on that probe. Thus, timepoints represent percentages of the probe period from onset to response.

PM costs were measured by first averaging the response time to correct OG task probes from non-PM trials separately from early and late experiment blocks at each difficulty level (6 values: easy/medium/hard × early/late). As in previous work from our lab (Koslov et al., 2019), PM costs for each probe were calculated by subtracting the corresponding non-PM trial OG task RT (depending on time and difficulty) from OG task RTs on probes from PM-trials. The early/late designation was used to account for a decrease in OG task RT that occurred across early to late experimental blocks (ß = −0.073, *t*(29) = −6.877, 95% CI [−0.084, −0.062], *p* < .001). After controlling for time-period effects by baselining the OG task RT separately for early and late blocks, PM costs were similar across the two experimental time periods (ß = 0.003, 95% CI [−.017, 0.010], *t*(29) = 0.248, *p* = .806).

In order to better describe and visualize the relationship between fixation patterns and PM costs, we extracted three PM cost bins: “no-cost,” “medium-cost,” and “high-cost” probes. No-cost probes were those where the PM cost was less than or equal to zero (~41% of probes per participant, mean = 260, *SEM* = 11). High-cost probes were those with a PM cost of at least 200 ms (~32% of probes per participant, mean = 207, *SEM* = 11), and probes with costs between the two extremes were designated as medium-cost probes (~27% of probes, mean = 176 probes, *SEM* = 6). These bins were selected so that we could include the meaningful cutoff of PM costs being ≤0 (interpreted in previous work as no evidence of active monitoring) as a bin, as well as having a high-cost bin with a similar number of probes to the no-cost bin and enough medium-cost probes to perform all analyses. Importantly, we observed probes that fell into each PM cost bin across all participants and difficulty levels. Additionally, previous work using PM costs has demonstrated that individuals adjust their PM strategy gradually over time in response to cognitive demands (Koslov et al., 2019). Here, we tested whether moment-to-moment shifts in PM costs occurred gradually over time or were better described as random. To do this, we compared the PM costs surrounding each probe (lag −3:+3), averaged across each PM probe bin and collapsed across difficulty level. Consistent with previous work suggesting gradual shifts in PM costs over time, we found that the surrounding PM costs fluctuated along with the central (lag = 0) PM cost (ß = 0.066, 95% CI [0.060, 0.071], *t*(29) = 12.166, *p* < .001).

### Statistical procedures

Unless specifically indicated in the results section, all subsequent analyses were performed only on probes where participants were asked to perform both the PM and OG tasks (PM trials). Previous work from our lab (Koslov et al., 2019), has demonstrated that OG task difficulty levels can be placed along a continuum from easy to hard, and so OG task difficulty was treated as a continuous predictor. When analyzing behavioral effects across task difficulty levels, we used standard mixed-effects statistics including relevant random effects of participant slopes and intercepts implemented using the lme package (Bates et al., 2015) in R and evaluated further using the lmerTest package (Kuznetsova et al., 2017). Example formula used for the interaction and main effect models are below:

$${\displaystyle \begin{array}{l}\boldsymbol{Interaction}: lmer\left( RT\sim pmType\ast difficulty+\left(1+ pmType\ast difficulty| Subject\right)\right)\\ {}\boldsymbol{Main}\ \boldsymbol{Effects}: lmer\left( RT\sim pmType+ difficulty+\left(1+ pmType+ difficulty| Subject\right)\right)\end{array}}$$

Terms within the inner parenthesis indicate random effects computed for each subject, while terms outside of the inner parenthesis indicate fixed effects. Exceptions to the above models were used when the data did not support the full specification of random effects included in the model. For PM accuracy, an intercept for each subject was used, but not a term for trial difficulty. For OG accuracy and cumulative-OG dwell times, separate random effect terms for PM type and difficulty were used, but not an interaction term.

When comparing cumulative dwell time measures to either PM costs or PM accuracy, we used a bootstrap permutation analysis (6,000 iterations) to assess population level reliability of effects (Efron, 1979; Kim et al., 2014; Lewis-Peacock et al., 2016). For the model comparisons, we also used the bootstrap modelling approach. We fit the models using all combinations of the eye-tracking measures (cumulative OG-dwell time, cumulative PM-dwell time, trial difficulty, and interactions), and then separately fit models with PM costs, trial direction, and the interaction. For each model, we extracted the weighted BIC (Vandekerckhove et al., 2015) and *R*^2^ values on each iteration of the bootstrap analysis. Importantly, when using models to explain variance in PM accuracy, all measures were recorded from probes preceding the PM-target appearance, not from the probe where a PM-target was displayed. We did this for two reasons. First, we were interested in evaluating the relationship between PM strategies and PM accuracy leading up to the noticing and acting on PM targets. Including the PM-probe itself would add extra variability introduced by other factors, like bottom-up noticing of the PM-target, rather than how the preceding strategy relates to accuracy. Secondly, PM costs cannot be calculated from probes where no OG task RT is collected, like in cases where a PM response is made, making a comparison between costs and dwell times impossible there.

To evaluate differences in fixation location across OG task difficulty levels and PM cost bins, fixations were first identified using SR EyeLink’s default parsing parameters. The average Euclidean distance from the center of the screen was used as the fixation distance from onset to offset of each fixation. Fixation onsets and offsets were scaled in the same manner as probe timescales and expressed as the percentage of probe period elapsed during that fixation. For example, on a probe with a response time of 1,000 ms, a fixation that began 200 ms after probe onset and ended at 400 ms after probe onset would be scaled as lasting from 20% to 40% of the probe period. Mixed-effect linear regression was then used to determine whether there was a relationship between fixation distance and PM costs at each timepoint. When evaluating fixation location differences, we used an FDR-correction method to assess statistical significance across each 2-s probe (corrected for 100 timepoint comparisons). FDR correction is a process to control for an acceptable number of false positives out of all statistically significant tests. This control is carried out by first ordering the *p* values from the original statistical test from smallest to largest. Then, the formula *p*_*i*_ < Alpha × (*i*/*N*) is used to determine where H_0_ should be rejected. Here, *i* is the ranked order of the *p* value, *N* is the total number of tests, and alpha = 0.01 or 0.05. Timepoints were binned into early (the first half of the probe period) and late (the last quarter of the probe period). Mixed effect linear regression was then performed to extract a *t* score describing the relationship between PM cost and fixation distance in each time bin for each participant. These by-subject *t* scores were then compared across OG task difficulties. Details of all statistical procedures and preprocessing steps can be found in the code provided online.

## Results

### Behavioral performance

Ongoing (OG) task accuracy was similar across face-target and scene-target trials, *F*(1, 29) = 1.23, *p* = .276, so we collapsed across them for all subsequent analyses. Prospective memory accuracy was not impacted by the difficulty level (easy/medium/hard) of the OG task (ß = 0.030, 95% CI [−0.065, 0.005], *t*(29) = −1.673, *p* = .105). Thus, individuals were not sacrificing PM accuracy for the OG task as it became more difficult. Next, we examined OG task accuracy as a function of task difficulty and trial type (PM/non-PM). OG task accuracy decreased as difficulty increased (ß = −0.120, 95% CI [−0.135, −0.106], *t*(29) = −16.211, *p* < .001). OG task accuracy was slightly lower on PM trials than on non-PM trials (ß = 0.015, 95% CI [−0.001, 0.031], *t*(29) = 1.839, *p* = .076), but there was no interaction between trial type and difficulty (ß = 0.002, 95% CI [−0.016, 0.019], *t*(29) = 0.207, *p* = .837).

**Fig. 2:**
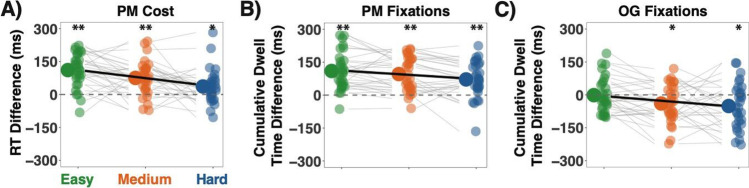
The difference between PM and non-PM trial types across difficulties for each measure. Big points represent the group mean, while smaller, faded points represent the average for individual participants at each difficulty level. Gray lines show the relationship for individual participants across difficulties, while the black line is the group average. The dashed line at zero on each plot indicates where the PM value of the measure was equal to the non-PM value. Positive values mean that the PM value was greater than the non-PM value. **a** Ongoing task response time difference between PM and non-PM trials (i.e., PM cost). **b** The difference in cumulative dwell time on PM-stimuli between PM and non-PM trials. **c** The difference in average cumulative OG-array dwell time for PM versus non-PM trials. **p < .01, *p < .05

We also examined OG task reaction times (RTs) as a function of task difficulty and trial type. There was a main effect of difficulty (ß = 142.62, 95% CI [126.50, 158.74], *t*(29) = 17.341, *p* < .001), with participants responding to the OG task more slowly as difficulty increased. There was also a main effect of trial type (ß = −76.25, 95% CI [−96.90, −55.59], *t*(29) = 7.235, *p* < .001), with participants responding to the OG task more slowly on PM trials compared with non-PM trials—this is the canonical effect known as “PM cost.” Having established a difference in OG task RT between PM and non-PM trials, we next investigated PM costs across difficulty levels. PM costs increased as OG task difficulty became easier (ß = 37.33, 95% CI [18.98, 55.67], *t*(29) = 3.988, *p* < .001; Fig. 2a), such that PM costs on average were greater than zero across all difficulty levels though to a greater degree for easy probes (μ = 111.19, 95% CI [82.15, 140.24], *t*(29) = 7.83, *p* < .001), than medium probes (μ = 75.45 , 95% CI [46.24, 104.66], *t*(29) = 5.28, *p* < .001), or hard probes (μ = 36.54, 95% CI [7.67, 65.42], *t*(29) = 2.59, *p* = .015). OG task RT and PM accuracy summaries can be found in Table 1.

**Table 1:** PM and OG task performance

Trial Type	OG task RT (ms)		PM cumulative dwell time (ms)		OG cumulative dwell time (ms)		PM accuracy	
Non-PM	Easy: Med: Hard:	885 (24) 1,086 (30) 1,224 (31)	Easy: Med: Hard:	52 (12) 52 (12) 60 (14)	Easy: Med: Hard:	738 (25) 928 (30) 1030 (33)	N/A	
PM	Easy: Med: Hard:	1,015 (24) 1,162 (27) 1,247 (27)	Easy: Med: Hard:	162 (15) 147 (14) 132 (13)	Easy: Med: Hard:	736 (22) 888 (28) 979 (31)	Easy: Med: Hard:	67.1% (4.0%) 63.5% (4.9%) 61.1% (4.3%)

### Eye-tracking results

To quantify monitoring behaviors, we analyzed the amount of time individuals gazed at the stimuli relevant for the OG task and at stimuli relevant for the PM task. Data from non-PM trials served as a baseline for PM trials. As expected, participants fixated longer on OG task stimuli as the difficulty of the OG task increased (ß = 133.60, 95% CI [114.42, 152.78], *t*(29) = 13.66, *p* < .001). However, participants fixated less on OG task stimuli on PM trials compared to non-PM trials (when the PM stimuli could be ignored; ß = 30.44, 95% CI [3.57, 57.32], *t*(29) = 2.221, *p* = .034), and this fixation deficit increased with task difficulty (i.e., interaction; ß = 24.76, 95% CI [6.19, 43.33], *t*(29) = 2.613, *p* = .014, see Fig. 2c). On the contrary, participants consistently fixated longer on PM stimuli for PM trials compared to non-PM trials (ß = 92.45, 95% CI [65.35, 119.56], *t*(29) = 6.686, *p* < .001), although this PM-fixation surplus shrunk as the OG task became more difficult (ß = 19.23, 95% CI [8.34, 30.12], *t*(29) = 3.462, *p* = .002, see Fig. 2b). In sum, these eye-tracking data generally corroborate the inference from the PM cost data that monitoring decreases as the OG task becomes more difficult.

We compared these measures directly and found that the link between PM costs and PM fixations was positive, but it weakened with increasing task difficulty, from easy (*R*^2^ = 0.169, 95% CI [0.133, 0.207]), to medium (*R*^2^ = 0.080, 95% CI [0.052, 0.105]), to hard trials (*R*^2^ = 0.036, 95% CI [0.021, 0.056]). The relationship between PM costs and OG task fixations was also positive (*p* < .001), but this coupling strengthened with increasing task difficulty (easy *R*^2^ = .345, 95% CI [0.282, .0.409], medium *R*^2^ = .468, 95% CI [0.412, 0.526], hard *R*^2^ = .504, 95% CI [0.445, 0.564], *p* < .001). Partial regression confirmed that even though OG task fixations and PM fixations were negatively correlated across probes, these two measures explained unique variance in PM costs (PM-fixations: ß = 0.62, 95% CI [0.57, 0.68], *p* < .001; OG-fixations: ß = 0.89, 95% CI [0.85, 0.93], *p* < .001). Together, PM fixations and OG task fixations accounted for approximately 78% of the variance in PM costs. The remaining variance is likely explained by differences in saccade timing and fixations that fell outside of either the PM or OG task regions.

### Unique profiles of monitoring across PM costs

**Fig. 3:**
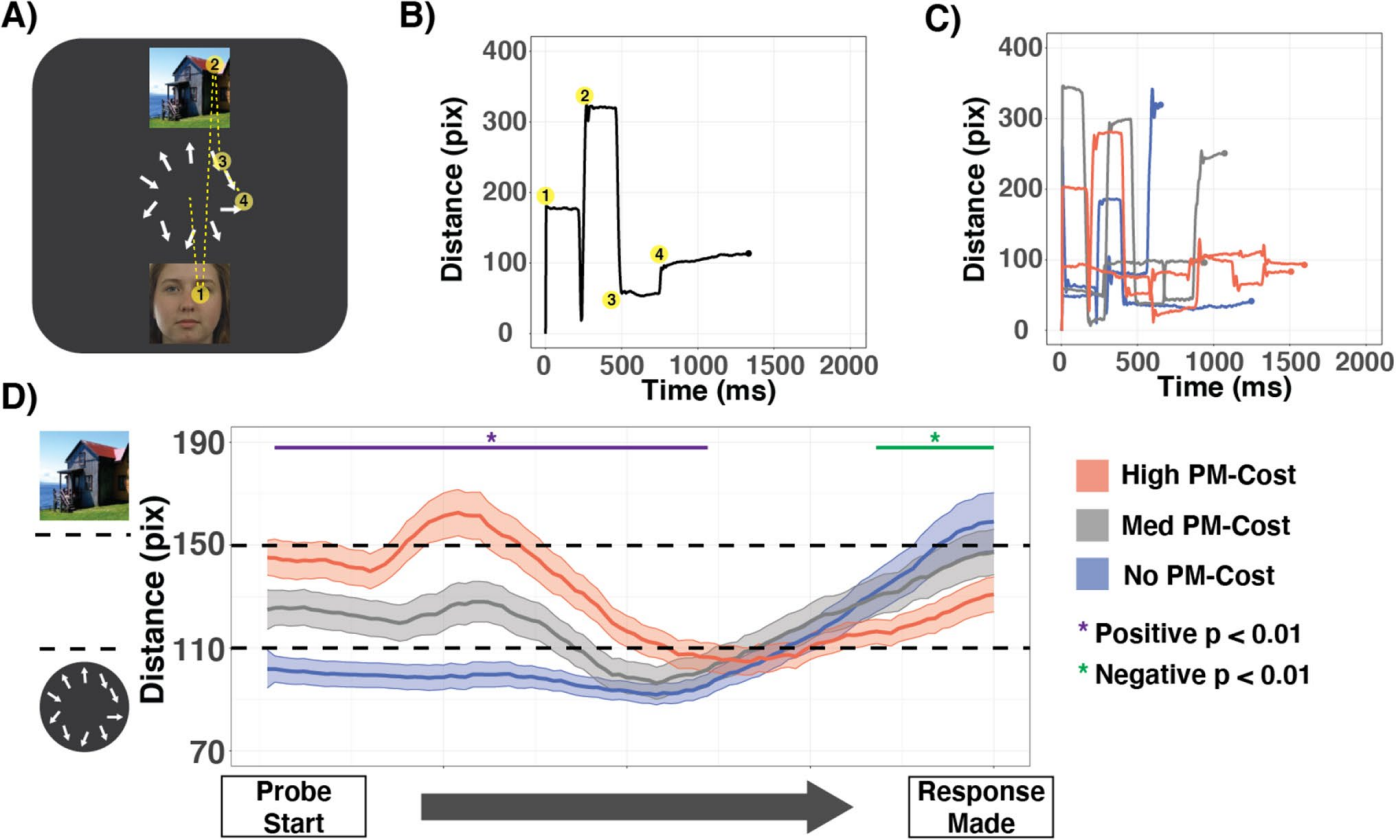
Fixations were tracked over time on each probe. **a** Fixation pattern for an example probe. Yellow circles represent individual fixations. **b** Fixation distance from center for this example. Yellow circles correspond to the start of fixations labelled in (**a**). **c** Fixation distances split by PM cost observed on a few sample probes. **d** Group-averaged fixation distances for easy probes split by PM cost, with a normalized *x*-axis to account for variable response times. Fixations that occurred less than 110 pixels from the center of the screen were within the OG arrow array, while fixations that occurred over 150 pixels from the center of the screen were towards PM-stimuli. The dashed lines in **d** represent these distance boundaries. The purple line indicates timepoints where there was a significantly positive relationship between PM cost and fixation distance from the center of the screen. The green line indicates where the relationship between fixation distance and PM cost was significantly negative. FDR correction for 100 timepoint comparisons was employed for calculation of *p* values. (Color figure online)

The presence of PM costs is commonly taken as evidence of proactive control, whereas the absence of PM costs is often interpreted as evidence of reactive control. We leveraged the temporal specificity of eye-tracking to evaluate the relationship between PM costs and fixation patterns across trials. To compare fixation patterns across trials with variable response times, we scaled the temporal axis of each probe as the percentage of time elapsed between the onset of the probe stimuli and the participant’s response. When PM costs were high (Fig. 3d, red), participants demonstrated early and prolonged fixations on the PM stimuli, followed by later OG task fixations. There was a main effect of PM costs over the first half of each probe, with fixations being further away from the center of the screen as PM costs increased (FDR corrected *p* < .01; significant time points for easy difficulty probes shown in Fig. 3d in purple). This result was similar for each OG task difficulty level where there was a main effect of PM cost bin over the first portion of each probe (negative *p*s < .01; easy: 2%–61% of trial, medium: 2%–56%, hard: 2%–27%). While fixation patterns during the first half of PM probes were similar across OG task difficulty levels, the average distance of fixations from the center of the screen decreased with increasing OG task difficulty (ß = −3.013, 95% CI [−5.063, −0.962], *t*(29) = −2.88, *p* = .007). Correspondingly, the main effect of PM cost on average fixation distance measured during the first half of probes decreased in magnitude as difficulty increased (ß = −29.778, 95% CI [−35.822, −23.734], *t*(29) = −9.657, *p* < .001).

When no PM costs were observed (Fig. 3d, blue), there was as expected no evidence for PM monitoring, at first. However, during the final quartile of the response period, participants shifted their gaze towards the PM stimuli. The average fixation distance during this fourth quartile was further from the center of the screen on probes where no PM costs were observed compared to probes with medium or high PM costs (FDR-corrected *p* < .01; significant time points for easy probes shown in green in Fig. 3d). This relationship was observed across all OG task difficulty levels (negative *p*s < .01; easy: 84%–100% of trial, medium: 74%–100%, hard: 79%–100%), and the magnitude of the relationship did not change as a function of task difficulty (ß = 0.544, 95% CI [−4.204, 5.291], *t*(29) = 0.224, *p* = .824). Although it is worth noting that PM fixations were not made on all probes. In particular, on probes where no PM costs were observed, participants were less likely to ever fixate on PM stimuli than on probes with medium or high PM costs (ß = 0.102, 95% CI [−0.122, −0.080], *t*(29) = −9.768, *p* < .001; no-cost: 57% *SE* = 4%; medium-cost = 47%, *SE* = 4%; high-cost = 37%, *SE* = 4%).

The above analyses highlight how fixation patterns towards PM and OG stimuli differ across PM cost bins on PM-trials. In order to more fully characterize differences in attention allocation across PM costs, we compared cumulative OG-dwell times on PM versus non-PM trials for each PM cost bin. As shown in Fig. 2, we found that participants overall spent less time fixating on the OG-array on PM compared to non-PM probes. However, this decrease in OG task engagement was only true for no-cost probes (μ = −174 ms, 95% CI [−201 ms, −146ms], *t*(29) = −12.913, *p* < .001). For medium-cost probes, there was no significant difference in time spent fixating on the OG task array between PM and non-PM probes (μ = −16ms, 95% CI [−44 ms, 12 ms], *t*(29) = −1.152, *p* = .259). Conversely, on high-cost probes, cumulative OG-dwell times were significantly greater for PM than non-PM probes (μ = 169 ms, 95% CI [132 ms, 206 ms]) *t*(29) = 9.366, *p* < .001). Thus, we observed significant differences in both PM target monitoring and OG-task engagement across levels of observed PM costs.

### PM accuracy is best explained by including eye-tracking

**Fig. 4:**
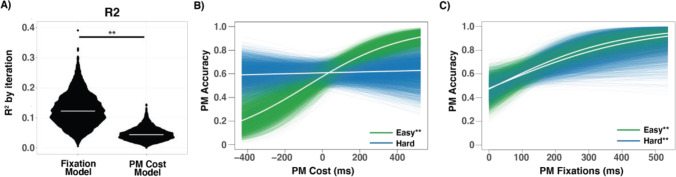
**a** The *R*^2^ value from each bootstrap iteration plotted for the best fixation model and the best PM cost model. The median *R*^2^ value (white lines) for the fixation model is approximately triple that of the PM cost model. **b** Depicts the relationship between PM cost (ms) and PM accuracy (predicted probability of a correct response) on each iteration for easy (green) and hard (blue) difficulty trials. Medium difficulty trials are not shown here but tended to have a relationship that fell between that of the easy and hard trials. **c** Plots the relationship between average cumulative PM-dwell times on each trial and subsequent PM accuracy (predicted probability of a correct response) from each bootstrap iteration. **p < .01. (Color figure online)

One discrepancy in previous research using PM costs is that this measure does not always relate to general PM performance. Here, we hypothesized that collecting eye tracking data to measure explicit monitoring behavior could help to bridge the gap between PM costs and PM accuracy. We evaluated different explanatory models to determine which factors best explained variance in PM task accuracy (see Methods). The best fitting model, as determined by weighted BIC, was the fixation model that included the main effects of OG fixations, PM fixations, and trial difficulty (wBIC median = 0.608, median *R*^2^ = .123, Fig. 4a). This model provided a significantly better fit than the model including only PM costs, trial difficulty, and their interaction (wBIC difference Wilcoxon pseudo-median: 0.586, 95% CI [0.576, 0.595], *p* < .001) and explained approximately triple the variance in PM accuracy than PM costs alone (*R*^2^ difference Wilcoxon pseudo-median: 0.079, 95% CI [0.078, 0.080], *p* < .001). Furthermore, adding PM costs to the best fitting model of fixations did not improve model performance (mean BIC difference: 47.064, 95% CI [46.725, 47.403], *p* < .001).

We found a positive relationship between PM costs and PM performance when the OG task was easy (mean β = 3.99×10^-4^, 95% CI [1.95*10^-4^, 6.33×10^-4^], *p* < .001, Fig. 4b), but this relationship disappeared when the OG task was hard (mean β = 0.16×10^-4^, 95% CI [−1.52×10^-4^, 1.75×10^-4^], *p* = .416), and the interaction between PM costs and these two difficulties was significant (mean ß = −1.87 , 95% CI [−3.26, -0.56], *p* = .003). The relationship between PM cost and PM accuracy on medium difficulty trials resembled the relationship that was observed on easy difficulty trials (mean β = 1.81×10^-4^, 95% CI [−0.12×10^-4^, 3.89×10^-4^], p = .034, not shown in figure). The relationship between PM accuracy and PM costs stands in stark contrast to what we found for cumulative PM-dwell times, which were positively correlated with PM accuracy across easy, medium, and hard difficulty trials (mean β easy = 5.66×10^-4^, mean β medium = 5.84×10^-4^, mean β hard = 5.00×10^-4^, all *p*s < .01, Fig. 4c). When modeled in isolation, OG fixations did not reliably predict PM performance at any difficulty level (all *p*s > .139). However, as noted above, the best model for explaining variance in PM accuracy included OG fixations as well as PM fixations and trial difficulty. Thus, it appears OG fixations provide subthreshold, but useful, information for explaining PM accuracy when included in addition to PM fixations and trial difficulty.

**Fig. 5:**
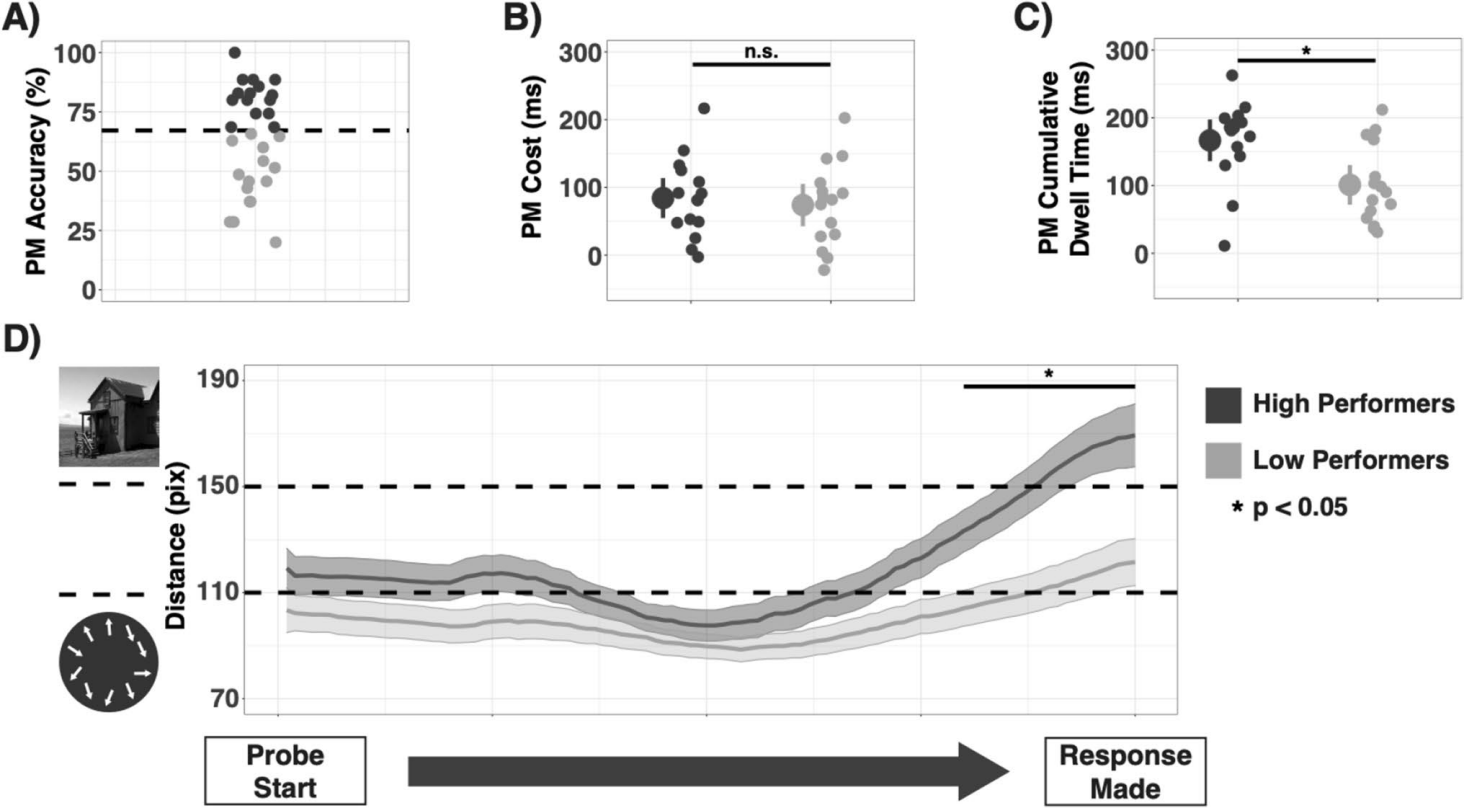
**a** Participants were median split by PM accuracy. High performers are depicted in dark gray; low performers are depicted in light gray. **b** PM costs were similar across both groups (small dots = individual subjects, big dots = group averages, and lines = 95% CI). **c** Cumulative PM dwell times differed between groups (*p = .005). **d** Fixation distance across time for high performers and low performers on probes where no PM costs were observed. *p < .05 (FDR corrected)

To characterize the relationship between PM accuracy and fixation patterns in more depth, we performed a median split analysis, separating participants into high and low performers on the PM task (median accuracy = 67%, group *N*s =15). The high performers had greater PM accuracy than the low performers across all difficulty levels (ß = 0.354, 95% CI [0.272, 0.437], *t*(28) = 8.407, *p* < .001, see Fig. 5a), with no interaction between difficulty and group (ß = 0.027, 95% CI [−0.098, 0.043], *t*(28) = −0.760 *p* = .454). Additionally, there were no differences between groups in OG task RTs (ß = 0.022, 95% CI [−0.084, 0.127], *t*(28) = 0.406, *p* = .688), or OG task accuracy (ß = 0.022, 95% CI [−0.037, 0.081], *t*(28) = 0.741, *p* = .465), nor differences in PM costs (ß = 10.24, 95% CI [−32.69, 53.18], *t*(28) = 0.468, *p* = .644, see Fig. 5b). There were no differences in cumulative OG-dwell times between groups (ß = 41.37, 95% CI [−58.78, 141.51], *t*(28) = 0.81, *p* = .425), but the high performers did have longer cumulative PM-dwell times (ß = −65.69, 95% CI [23.17, 108.21], *t*(28) = −3.028, *p* = .005, see Fig. 5c). Both groups had quite similar patterns of fixations. However, on no-cost probes, the high performers made more PM fixations immediately before making a response (*p* < .05, FDR corrected 80%–100% of probes, Fig. 5d).

## Discussion

We used eye-tracking to characterize active monitoring strategies for prospective memory and to evaluate how monitoring relates to the widely used measurement of PM costs. In general, high PM costs reflect a prioritization of the prospective intention with early, frequent, and prolonged monitoring of PM stimuli. Low PM costs are associated with less monitoring overall with an increase in late, brief checks of the PM stimuli. Our results suggest that PM costs do not reflect a monolithic measure of overt monitoring for PM stimuli. Rather, PM costs reflect different profiles of monitoring behaviors depending on task demands. PM costs were most tightly coupled with monitoring when the OG task was easy. As the task became harder, PM costs decoupled from monitoring and instead increasingly reflected fixations on the OG stimuli. This suggests that PM costs are driven by different sources—the level of monitoring for PM stimuli or engagement with the OG task—depending on the demands of the task. Together, this set of results supports the idea suggested by Ball and Brewer (2018), that individuals adjust the *nature* of monitoring in order to respond to the demands of the environment, rather than simply the frequency of monitoring. Returning to our question from the beginning of this paper, our findings suggest that remembering to stop at a delicious roadside stop for “the best bar-b-que” in the state will be accomplished by different mnemonic strategies, and that the degree to which individuals rely on these strategies is linked to cognitive load (e.g., the density of highway traffic and the intensity of backseat tantrums).

We also found that fixation measures explained a significant portion of variance in PM accuracy. More time spent fixating on PM stimuli was related to better PM accuracy, and high-performing participants demonstrated more monitoring, especially when there were no observable PM costs. Measurements of PM fixations were also better predictors of PM accuracy than were PM costs. When OG task demands were low, eye-tracking confirmed that the link between PM costs and PM monitoring was strongest and, in these situations, that PM costs were also positively correlated with PM accuracy. Strikingly, when OG task demands were high, there was no relationship between PM costs and PM accuracy. As OG task difficulty increased, eye-tracking revealed that PM costs corresponded less to monitoring of PM stimuli and more to greater engagement in the OG task, which is less diagnostic of performance on the PM task. These results suggest that the ubiquitous and useful measurement of PM costs may be inadequate to understand PM performance in more demanding (i.e., ecologically valid) environments in the real world, and they highlight the advantage of including spatiotemporally precise methods like eye-tracking.

According to the DMPV theory of PM, the absence of PM costs without an accompanying drop in PM performance is typically interpreted as an indication that participants are using a spontaneous retrieval strategy (e.g., McDaniel & Einstein, 2007). The pattern of fixations observed during periods without PM costs in the present study (prolonged early fixations on the OG task stimuli and brief late fixations on the PM stimuli) could be consistent with this interpretation. Individuals may have been using a strategy called “discrepancy plus search” (Lee & McDaniel, 2013; McDaniel et al., 2004), in which one perceives some discrepancy in the environment (or in memory) that automatically triggers an effortful search to find the source of that discrepancy. As the PM stimuli were peripherally available to individuals while they performed the OG task, they may have been able to rely on this strategy. Additionally, probes where no cost was observed were likely to contain no overt fixation on the PM stimuli. However, there was also evidence for a decrease in cumulative OG dwell times on no-cost probes, suggesting that individuals may have sometimes been implementing an effortful tradeoff between OG and PM attention allocation. It is likely that reactive and proactive strategies underlie subsets of no-cost probes in this and previous experiments, and by using time-sensitive eye-tracking measures we can move towards more precisely identifying when each strategy may be engaged at any given moment.

Previous PM studies using eye-tracking have consistently found that monitoring for PM stimuli is positively correlated with PM success. Shelton and Christopher (2016) found that when individuals were reminded of a PM intention by semantically related cues, they were more likely to monitor for and complete that intention. Bowden et al. (2017) found that participants who were informed about the temporal proximity of a PM event increased monitoring as time approached, and this improved memory. In line with these findings, we observed that the more time individuals spent monitoring for PM stimuli prior to an event, the more likely they were to accomplish the PM task. Further analysis also revealed that high performers spent more time monitoring than low performers, but critically the PM costs observed for these groups did not differ. It is unclear whether the increase in late monitoring for high-performing individuals was related to increased proactive ‘checking’ or an increased influence from peripheral stimuli to drive attention allocation. Nonetheless, these results highlight the benefit of collecting direct measures of monitoring, rather than relying solely on indirect PM costs, to begin gaining a better understanding of the link between strategy and performance in PM and potential sources of group differences in PM performance.

Incidentally, fixating on PM stimuli does not guarantee that the PM target will be identified. The target was missed on 26% (*SEM* = 4%) of trials when the PM stimuli were fixated, though misses were characterized by shorter cumulative PM-dwell times than hits (mean difference = 103 ms, 95% CI [4 ms, 202 ms], *p* = .043). This result is in line with previous work that observed participants fixated on the PM target stimulus on up to half of trials in which they failed to perform the prospective intention (Hartwig et al., 2013; West et al., 2007). While participants did make occasional correct PM responses on probes where no fixation fell on PM-stimuli (μ = 1.47/participant), the low frequency of these PM responses makes it difficult to draw strong conclusions about no-fixation PM responses. The fixation tracking methods used here are useful but limited, as they are incapable of differentiating the quantity of monitoring (indicated by dwell times) from the quality of monitoring (related to capacity for processing the OG task and PM stimuli). However, despite the rate of no-fixation correct PM responses being low, it was significantly greater than the average rate of false alarm responses (μ rate difference = 3.29), *t*(29) = 2.67, 95% CI [0.77, 5.82], *p* = .01.

In addition to increasing our understanding of PM performance across different levels of cognitive demands, the use of eye-tracking can also increase our understanding of the processes that underlie the commonly used PM costs measure. Currently, there are two primary theories describing the mechanisms that lead to PM costs. The DMPV (Scullin et al., 2013; Shelton & Scullin, 2017) describes attentional resources as shared between the PM and OG tasks. According to this view, it is the sharing of resources between the two tasks that produces either PM costs (when the PM task receives relatively more resources) or PM forgetting (when the OG task receives relatively more resources). In contrast, the delay theory of PM (Heathcote et al., 2015) posits that PM costs primarily stem from a shift in the OG task decision threshold rather than shared-resource constraints. This theory, derived primarily from evidence-accumulation modeling of aggregate RTs, predicts that PM costs arise not because processing capacities for OG tasks differ between PM and non-PM contexts (as would be predicted by shared-resource accounts), but because individuals act more conservatively when they perform both tasks (Strickland et al., 2018).

The patterns of fixations that we observed suggest that both theories may be related to PM costs, but in different situations. Previous work in decision making has demonstrated that fixation durations are related to evidence accumulation rates (Armel et al., 2008; Krajbich et al., 2010). In support of the shared-resource DMPV account, PM costs were best described by both PM-fixation and OG-fixation measures, indicating that an explanation of costs relying entirely on more time spent accumulating evidence (because of increased thresholds) for the OG task is insufficient. Additionally, on no-cost probes, there was an observed sacrifice in OG task dwell times to accommodate PM task monitoring. These observations point to a shared attentional capacity mechanism being used during PM-task performance.

However, there were also patterns that could be interpreted as supporting a delay theory to PM costs. First, on high-cost probes, individuals most often fixated on the PM-stimuli before switching to the OG task. This pattern of fixations could be described as an early delay or non-decision process, though the duration of the early fixations observed here is significantly longer than suggested in delay modeling work (Strickland et al., 2019b). Additional support for a delay model is that on no-cost probes, fixations to OG task stimuli were dominant, and only sometimes accompanied by brief checks of the PM stimuli near the end of the response window. This could be consistent with the delay theory of PM as it possibly reflects a process by which participants accumulated evidence for an OG task decision, but then delayed their response until they had glanced at the PM stimuli. The mixed evidence in support of both theories of PM costs may be because the task design used here requires greater levels of cognitive control than other paradigms. For example, both Boag et al. (2019) and Strickland et al. (2019a) found that PM costs were related to both changes in evidence accumulation rates and decision thresholds when the OG task was performed in cognitively demanding or time-sensitive environments. Additionally, we observed that the relationship between PM costs and OG and PM cumulative gaze times shifted in response to different levels of ongoing demands. This suggests that the underlying mechanics that contribute to PM costs also shift as individuals adapt to environmental demands.

Prospective remembering is a complex and multifaceted process. Individual differences in working memory capacity, updating, task switching, and inhibitory control can factor into PM performance (Ball & Brewer, 2018; Ballhausen, et al., 2019; Koslov et al., 2019; Rose et al., 2010; Zuber et al., 2016; Zuber et al., 2019). Unexplained variance in prospective remembering can likely be attributed to some combination of these processes. Models of multitasking have attempted to formalize the underlying mechanisms necessary to accomplish such behavior (e.g., Broeker et al., 2018; Salvucci & Taatgen, 2008; Wickens, 2008). It is possible that the fixation patterns observed here represent individuals balancing focal versus peripheral attentional resources in an attempt to maximize multitasking ability (e.g., Wickens, 2008), or that shifts in the timing of PM fixations relate to changes in multithreading policies individuals adopt (e.g., Salvucci & Taatgen, 2008). While an in-depth discussion of the relationship between theories of multitasking and the overlap with prospective remembering is beyond the scope of this manuscript, we believe that eye-tracking is a promising avenue for differentiating between the ways individuals adjust attention allocation strategies in support of performing prospective and ongoing intentions simultaneously. In addition to individual differences in cognitive control and multitasking abilities, differences in how prospective intentions are encoded vary across individuals and can have substantial impacts on PM strategies and performance (McDaniel & Scullin, 2010; McFarland & Glisky, 2012; Scullin et al., 2018). The current experiment did not differentiate between attentional and retrieval components of prospective remembering that most likely operate as connected, but distinct, processes (e.g., Meier & Zimmermann, 2015). Our findings add to a growing body of literature that suggests that additional factors beyond PM costs should be considered when evaluating PM performance. While the use of eye-tracking can provide a time-sensitive measure of overt monitoring, future work would benefit from larger samples and the measurement of individual differences in cognitive control abilities that contribute to PM (e.g., Zuber et al., 2019). Additionally, research on working memory (Mathôt, 2020) and prospective memory (Moyes et al., 2019) suggests that pupil size is responsive to cognitive load. Future eye-tracking work could greatly benefit from the combination of both pupillometry and fixation tracking to better describe how the maintenance of and monitoring for prospective intentions are carried out in different environments.

Prospective memory tasks are generally characterized by the formation, retention, and subsequent execution of a prospective intention (Kliegel et al., 2011; Rummel & McDaniel, 2019). In the real world, delays between the formation and execution of prospective intentions vary from seconds to many days or even weeks long. The delay intervals in the present experiment ranged from 2 to 30 s, which are shorter than in many other prospective memory experiments. Previous research has suggested that PM costs and PM performance may decrease after an initial brief (~2 min) period (Brandimonte & Passolunghi, 1994; McBride et al., 2011), and this could limit the generalizability of our results. The monitoring strategies described here may most aptly apply to specific periods of monitoring that occur after PM interruptions (Dismukes, 2012; Dodhia & Dismukes, 2009) or after external reminders (e.g., Scullin et al., 2013; Shelton & Christopher, 2016). However, it is unclear whether the length of the retention interval influences how individuals refresh thoughts about PM intentions (Mahy et al., 2018; Scullin et al., 2018). An important direction of future research will be to test how the mechanisms associated with monitoring, including measurements of fixation patterns and PM costs, may change over increasingly long delay periods.

### Concluding remarks

In summary, we used eye-tracking to measure monitoring behaviors in a PM experiment with varying cognitive loads. We found that PM costs do not reflect a monolithic monitoring process, but rather they are influenced by variations in timing, duration, and order of overt monitoring of task-relevant information. The work presented here represents a promising step forward in identifying the characteristics of cognitive control supporting PM and how the engagement of these processes change as individuals orient themselves to external demands.

## Data Availability

The data and code for the current study are available on osf.io (https://osf.io/jr5mv/).
